# IGRT versus non-IGRT for postoperative head-and-neck IMRT patients: dosimetric consequences arising from a PTV margin reduction

**DOI:** 10.1186/1748-717X-7-133

**Published:** 2012-08-08

**Authors:** Michael Schwarz, Kristina Giske, Armin Stoll, Simeon Nill, Peter E Huber, Jürgen Debus, Rolf Bendl, Eva M Stoiber

**Affiliations:** 1 Department of Medical Physics in Radiation Oncology, DKFZ INF 280, Heidelberg, Germany; 2 Department of Radiation Oncology, DKFZ INF 280, Heidelberg, Germany; 3 Department of Radiation Oncology, INF 400, University Hospital Heidelberg, Heidelberg, Germany; 4 Heilbronn University, Medical Informatics, Max-Planck-Str. 39, Heilbronn, Germany

**Keywords:** Head-and-neck cancer, Adaptive radiotherapy, Image-guided radiation therapy, Correction strategies, Dose re-calculation

## Abstract

**Background:**

To evaluate the impact of image-guided radiation therapy (IGRT) versus non-image-guided radiation therapy (non-IGRT) on the dose to the clinical target volume (CTV) and the cervical spinal cord during fractionated intensity-modulated radiation therapy (IMRT) for head-and-neck cancer (HNC) patients.

**Material and Methods:**

For detailed investigation, 4 exemplary patients with daily control-CT scans (total 118 CT scans) were analyzed. For the IGRT approach a target point correction (TPC) derived from a rigid registration focused to the high-dose region was used. In the non-IGRT setting, instead of a TPC, an additional cohort-based safety margin was applied. The dose distributions of the CTV and spinal cord were calculated on each control-CT and the resulting dose volume histograms (DVHs) were compared with the planned ones fraction by fraction. The D50 and D98 values for the CTV and the D5 values of the spinal cord were additionally reported.

**Results:**

In general, the D50 and D98 histograms show no remarkable difference between both strategies. Yet, our detailed analysis also reveals differences in individual dose coverage worth inspection. Using IGRT, the D5 histograms show that the spinal cord less frequently receives a higher dose than planned compared to the non-IGRT setting. This effect is even more pronounced when looking at the curve progressions of the respective DVHs.

**Conclusions:**

Both approaches are equally effective in maintaining CTV coverage. However, IGRT is beneficial in spinal cord sparing. The use of an additional margin in the non-IGRT approach frequently results in a higher dose to the spinal cord than originally planned. This implies that a margin reduction combined with an IGRT correction helps to maintain spinal cord dose sparing best as possible. Yet, a detailed analysis of the dosimetric consequences dependent on the used strategy is required, to detect single fractions with unacceptable dosimetric deviations.

## Background

The use of intensity modulated radiation therapy (IMRT) in head-and-neck cancer (HNC) patients is known to be beneficial in terms of the organ at risk (OAR) dose sparing and as a result reducing once common toxicity. The quality of life after treatment can be increased by preserving critical organ function such as salivation and swallowing [1].

In fractionated therapy, however, patient positioning uncertainties and interfractional anatomical changes may limit the advantages of IMRT, e.g. if OARs shift into the high-dose region. As a consequence, the OAR can receive higher doses than originally prescribed [2,3]. For that reason safety margins need to be added to the clinical target volumes to keep the tumor control probability high. These margins enlarge the high-dose region and thus worsen the dose distribution from the OARs point of view, since the distance between the avoidance structures and the dose gradients of the high-dose region is decreased. Further interfractional variations in the position of the OAR, which are known to occur in spite of elaborate patient fixation devices [4-6], may also lead to differences in the applied dose distribution.

To overcome these setup problems, image guidance can be used to correct for patient positioning errors prior to irradiation. Currently, rigid corrections can only be realized in daily practice by the use of a target point correction (TPC). Still, a reduced safety margin is required to account for positioning uncertainties of technical equipment and patient anatomy, e.g. arising from deformations that cannot be corrected by a TPC. This margin cannot be omitted even if daily image guidance is used. Yet, the magnitude of this remaining margin is still under investigation [7].

A reduction of the safety margins, which account for rigid interfractional variations, is promising, since even small margins can result in a large additionally irradiated volume [8]. This leads to an increasing amount of target surrounding normal tissue being unwantedly exposed to the prescribed high dose. Additionally, the bigger volume of the high-dose region results in an increased probability for the nearby OARs to move into these regions.

In spite of the advantages of IGRT, it needs to be kept in mind that an approach with reduced margins requires continuous imaging along the treatment course. Whether the drawback of the additional low dose but large volume exposure of the imaging itself, and the prolonged treatment time outweigh the advantage to reduce dose to the volume surrounding the clinical target volume (CTV) is still of major interest for daily routine. So to clarify the benefit of IGRT, an individualized analysis of the dose distributions arising from different patient correction strategies during the treatment course is needed. A previous study evaluating the actual delivered dose to the parotid glands showed no significant difference in parotid sparing capability between an IGRT and non-IGRT approach [9]. Comparison of the capability to spare other organs at risk and to maintain the CTV coverage in the presence of deformations is still an open issue.

To answer this remaining question, this study focuses on the dose coverage of the CTV and on the possibility to maintain the planned dose of the spinal cord during fractionated radiotherapy. Therefore a margin-based non-IGRT approach is compared with a margin-reduced IGRT approach. For the IGRT approach a TPC is used which is derived from a registration closely fitted to the high-dose region to achieve optimal PTV coverage. Especially for the spinal cord it is important to clarify to which extent the reduced distance between the cervical cord and margin-enlarged high-dose region is disadvantageous in the non-IGRT setting. A fraction-by-fraction analysis of 4 HNC patients is presented to exemplarily show the variety of possible dosimetric effects arising from positioning variations and deformations.

## Methods and Material

### Patients

Four HNC patients with daily kilo-voltage control-CT scans were chosen for this retrospective analysis; written informed consent was obtained from all patients prior to inclusion. All patients received postoperative radiation therapy with an integrated boost technique in combination with cisplatin-based chemotherapy at the German Cancer Research Center (DKFZ). Three patients were treated for oropharyngeal and one for hypopharyngeal cancer (patient 4). IMRT was performed using a 6 MV linear accelerator (Siemens Artiste) combined with an in-room on-rail spiral CT-scanner. A total of 118 (28–32 per patient) CT-scans (0.98 × 0.98 × 2.0 respectively 3.0 mm^3^) were evaluated. Re-planning on the CT-scans in case of large positioning deviations was consciously not considered in this study, since daily re-planning is yet very time-consuming in clinical routine.

For patient immobilization an individually customized fixation device, composed of a scotch-cast mask attached to a vacuum mould, was used. Patients were positioned using a stereotactic setup. Both, fixation and positioning setup have been described previously [4].

### IMRT plans and margins

In all 4 patients 2 CTVs were defined. For better differentiation of the 2 CTVs in the integrated boost technique, the CTV including the cervical and supraclavicular lymph nodes is denoted extended CTV (eCTV). The second CTV which encompasses the previous gross tumor region only is denoted CTV. The spinal cord was delineated on the planning-CT and an additional standardized virtual contour was defined, which encircles the cord by 7 mm to control the dose gradients in its immediate neighborhood. Total dose prescriptions were 70.4 Gy to the CTV (single dose 2.2 Gy) and 57.6 Gy to the eCTV, while the maximum dose to the spinal cord was limited to maximal 40 Gy.

Two different IMRT plans were generated to clarify the impact of the use of IGRT vs. non-IGRT on the cord dose: The first plan is the IGRT plan which was applied in clinic using daily image-guidance. Both CTVs, the eCTV and the CTV, were extended by a 3 mm CTV-to-PTV margin to cope for remaining uncertainties, e.g. uncertainties for applying the TPC, for deriving the vector from the registration process, and for the remaining uncertainties caused by deformations. First estimations suggest that a 3 mm CTV-to-PTV margin is sufficient [7]. For the non-IGRT scenario a second plan was generated in which both CTVs were enlarged with the 3 mm CTV-to-PTV margin and an additional margin deduced in accordance to the van-Herk recipe [10]. This margin was applied to compensate for rigid interfractional positioning variations. It is deduced from a cohort of 45 patients. The additional margin amounts for 4 mm [4]. The margin is derived from local positioning uncertainties of the CTV region, however, in this study it is also applied to the eCTV to approximate the actually required margin. This may lead to an underestimation of the size of the margin needed for the eCTV, since the large eCTV is subject to deformations to a bigger extent. Therefore, in this study only the CTV is considered in the dose analysis.

### Target point correction for IGRT

To simulate the dosimetric effects on the cervical cord and CTV the IGRT plan is used and a TPC is applied on each control-CT. The required correction vector for the TPC was determined using rigid registration based on a small registration box which encapsulated the CTV only. Translational and rotational errors were calculated, however, since no hexapod table was available only the translational errors were corrected for in the TPC. Yet, the rotational errors were mostly smaller than 1°. The advantage of the focused registration - in contrast to an IGRT correction scenario using a large registration box - is a better alignment of the CTV throughout the treatment course, where the gross tumor was previously located. Its drawback is a worse placing of more distant structures like the cervical cord in case of deformations, which might have an impact on spinal cord dose. For the rigid registration an algorithm based on mutual information was used [4,11]. The accuracy of the registration method was previously quantified to be 0.2 ± 0.1 mm [4].

In contrast, in the non-IGRT setting, the treatment plan including the additional safety margin is applied to all control-CTs without a TPC.

### Estimation of the position of the CTV and spinal cord on the control-CTs

To calculate the DVH for each fraction, re-contouring of the CTV and spinal cord on each control-CT is a prerequisite.

To estimate the actual position of the CTV, a rigid registration of the corresponding control-CT with the planning-CT is performed using the registration box which encompasses the CTV. Afterwards the CTV contour is rigidly transformed according to the result.

To generate the new spinal cord contours a quasi elastic approach was applied. In this process each spinal cord contour is propagated within the same CT-slice in xy-plane (x: right-left, y: anterior-posterior) using rigid registration. Because of the independent slice-wise propagation, the actual position and bending of the spinal cord is updated. Subsequently all cervical cord contours were reviewed by one physician and manually adjusted if necessary.

Dose accumulation throughout treatment was not performed using voxel tracking, since the required deformable registration methods are currently still accompanied with unknown geometrical accuracy which can have an impact on dose re-calculation [12,13]. Thus all DVHs are presented fraction-by-fraction.

### Illustration of the results

**Figure 1:**
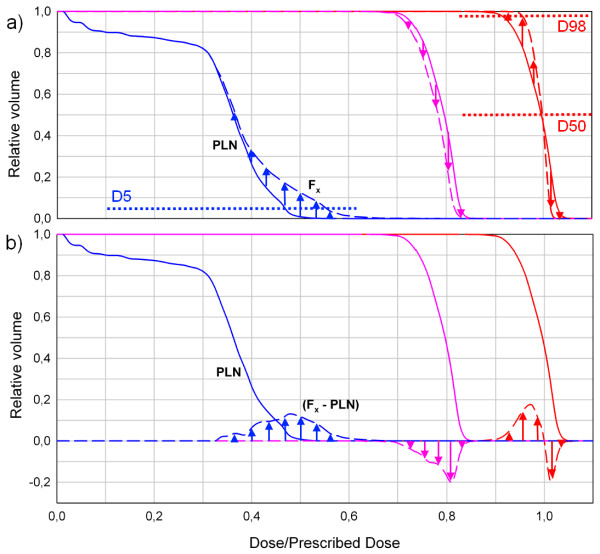
**Schematic diagram for DVH representation to ease comparison of the fraction DVH with the respective planning DVH for three exemplary curve progressions: The blue curve is representing an arbitrary organ at risk, the pink and red curves target volumes.** PLN: planned DVH. Fx: fraction x. A positive curve progression (e.g. indicated by blue arrows) implies that more volume of this organ at risk, e.g. spinal cord, receives the respective dose and vice versa.

The planned DVHs of the spinal cord and the CTV are plotted in one diagram. The planned DVH is subtracted from the DVHs generated for each fraction (Figure 1). These differences are plotted in the same diagram (Figure 1b). This type of diagram is used because it better illustrates little dose deviations compared to a set of all DVH curves (Figure 1 a). A positive curve progression implies that more volume of the spinal cord receives the respective dose and vice versa. For better comparison with the planning situation, all fraction prescriptions were scaled to the originally prescribed dose of 70.4 Gy. In the following all DVHs are plotted as demonstrated in Figure 1b.

**Figure 2:**
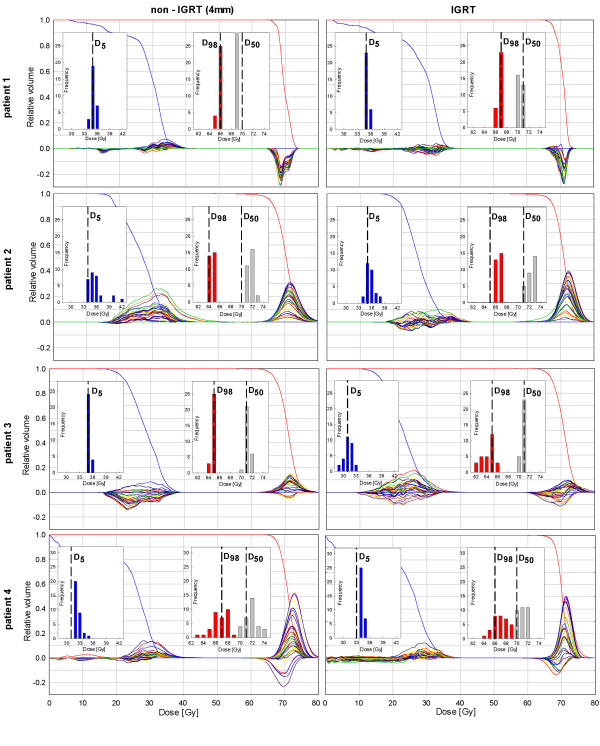
**Planning DVHs of the spinal cord (blue) and CTV (red) and respective fraction DVHs (plotted as illustrated in Figure**1**b) for the non-IGRT and the IGRT approach.** Additionally shown are the D5 (blue), D98 (red), and D50 (grey) histograms to demonstrate the variation over all fractions. The respective planned values are plotted in black dashed lines.

Additionally presented are the D5 values, which represent the dose variation to 5% of the volume of the cervical cord receiving the highest dose in all fractions. CTV coverage quality is represented by the D50 and D98 histograms. These histograms represent the dose variation along the treatment course of 50%, respective 95% of the volume of the CTV and are plotted in Figure 2.

## Results

The effects of the use of a TPC (IGRT approach) vs. the use of a non-IGRT approach on dose to the cervical cord and CTV are reported in detail. Figure 2 shows the diagrams with the respective DVHs. The DVHs of the spinal cord and the CTV are plotted in the same diagram to understand their interplay dependant on the used strategy.

Viewing the diagrams of all 4 patients it becomes apparent that the spinal cord receives doses differently to the originally planned ones even if IGRT is used. This happens despite an optimal alignment of the CTV and can be explained by deformations of the head-and-neck anatomy.

Patient 1: The CTV of this patient is the smallest of all patients. Considering the spinal cord dose, the DVHs of both approaches show no remarkable difference, this can also be seen in the D5 histograms in Figure 2. In terms of the CTV, again, both approaches result in similar dose coverage. Yet, the D50 histograms show a slightly better sustainment of the planned dose using IGRT.

**Figure 3:**
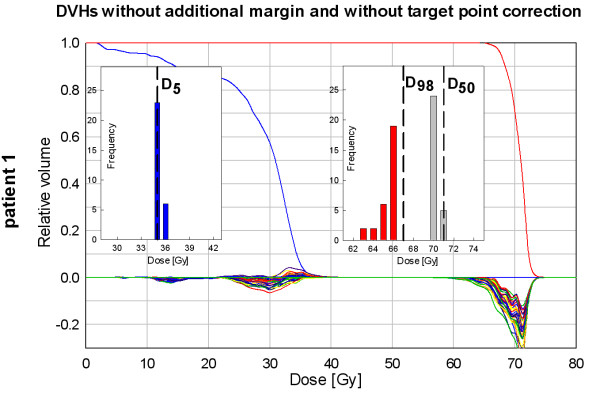
**Same representation as in Figure**2**, exemplarily showing the dosimetric changes in case of neither a target point correction (IGRT approach) nor a margin application is used in patient 1.**

In addition exemplarily presented for this patient is the data showing the dosimetric changes if neither a TPC nor an approach with an additional margin is used (Figure 3). Here, the D98 histogram indicates a worse CTV coverage compared to the planning situation in all fractions.

Patient 2: In this patient the CTV shrank over the treatment course due to a decrease of postoperative edema. Subsequently the contour of the CTV was manually adjusted on all control-CTs after the rigid transformation of the initial CTV contour. The effect of this volume decrease can be observed in both scenarios by the increasing positive curve progressions of the CTV, which results in a very good CTV coverage. Thus the D50 and D98 histograms do not show an underdosage in both settings.

The entire volume of the cervical cord receives more dose in the non-IGRT scenario, which can be seen by the curve progressions of the DVHs plotted in Figure 2. In both approaches the D5 of the cord receives more dose than planned. However, using IGRT, the variance of the D5 distribution is reduced and the distribution is shifted towards the planned value.

Patient 3: This patient showed only few and small geometrical variations of the CTV.

Looking at the D50 and D98 histograms, it becomes apparent that, on the one hand the dose to 50% of the CTV is sustained regardless of the strategy. On the other hand the delivered dose to 98% of the CTV is slightly smaller in one-third of the fractions compared to the planned situation using IGRT. The D5 histogram shows that the planned cervical cord dose can be better sustained over the treatment course in the non-IGRT setting.

Patient 4: This patient showed the largest absolute positioning deviations of the 4 patients. The results show that the D50 values are comparable in both strategies. However, the D98 histograms reveal that the CTV receives less dose than planned in 14 fractions in the non-IGRT approach compared to only 4 fractions using IGRT. In both strategies, the coord more dose than planned in all fractions (D5 histogram in Figure 2).

## Discussion

Aim of this study was to clarify whether IGRT with daily CT scanning can improve the quality of patient treatment in terms of cervical spinal cord sparing and CTV dose coverage in HNC patients compared to a non-IGRT approach. In this study “CTV” denotes the small part of the target volume with the prescribed dose of 70.4 Gy.

If no IGRT strategy is used, an additional margin needs to be applied to account for rigid positioning variations in order to assure CTV coverage [10]. This margin was determined in accordance to the van-Herk recipe for our HNC patients in a previously performed investigation, which analyzed the local geometrical uncertainties in a large HNC patient cohort [4]. The resulting margins of this former analysis were applied in this study.

In summary, this analysis reveals advantages and disadvantages for both, the IGRT and the non-IGRT approach:

Foremost it becomes apparent, that the cord never exactly receives the initially planned dose along the treatment course, even if IGRT is used. This is the case in all patients and can be explained by deformations of the head-and-neck anatomy. The deformations result in an imperfect alignment of the cord, since the applied TPC corrects for rigid positioning errors only and additionally occurring deformations cannot be adjusted. Using IGRT, the range of the D5 values varied between the patients from 2 to 9 Gy over the treatment course. This corresponds to a range of 3 to 13% related to the prescribed dose of 70.4 Gy.

Further noticeable is, that also the dose coverage of the CTV can sometimes not be sustained as planned using IGRT, even though only in one patient. This happens despite the use of a focused positioning correction. The correction vector for the TPC was derived from a registration of a small box surrounding the CTV, to achieve an optimal CTV coverage throughout the treatment course. Again, the impaired dose coverage might be explained by deformations of the head-and-neck anatomy resulting in changes of the dose distribution. In this context, it is important to allude that one should be cautious using the gross tumor volume as an alignment target, since asymmetric tumor shrinkage can affect the geometric relationship of the gross tumor volume to the OARs.

As expected, in the non-IGRT setting the cord often receives more dose than planned, which is readily identifiable in the curve progressions of the DVHs (e.g. in patient 2, Figure 2). The applied additional safety margin results in a smaller distance between the cervical cord and the dose gradient of the PTV. Thus the probability of the cord moving into the high-dose region in case of positioning changes increases. So the results show that the non-IGRT approach with margin-enlarged CTVs can be inferior in terms of cervical cord sparing compared to the IGRT approach. The extra volume covered by the additional margin surrounding the CTV is non-negligible, with an increase of 25-73% of the initial CTV in our patients.

It should also be kept in mind that tumors can regress during radiotherapy [14], especially if protocols with several weeks of sequential daily treatment are applied. In our analysis one patient showed shrinkage of the CTV during treatment due to a decrease of postoperative edema. This resulted in improved dose coverage of the CTV -- with and without IGRT -- but also implied more dose to the surrounding larger target volume of the neck. This is possibly undesired and can be avoided only if re-planning is performed. Yet, especially daily re-planning is very time consuming, though first concepts for daily fast re-planning are suggested by Ahunbay et al [15].

Additionally data for one exemplarily patient is presented which show the dosimetric effects if neither IGRT nor a margin approach is used. This approach, as expected, proved to be the worst scenario in terms of CTV coverage and thus is no serious treatment option.

## Conclusion

The present study confirms that IGRT is beneficial in terms of spinal cord sparing in the presence of interfractional motion. This is achieved by a margin reduction of the PTV which results in a bigger distance between the cervical cord and the steep dose gradients.

Demonstrating the benefit of IGRT in terms of spinal cord sparing, it also needs to be assured that IGRT, despite reduced margins, allows the same CTV coverage as a non-IGRT approach. Our study demonstrates that this can be achieved by a target point correction derived from a rigid registration with a registration box closely fitted to the high-dose region. However, detailed analysis reveals individual differences in the dose coverage of the CTV among the patients and strategies, worth inspection.

## Competing interests

The authors declare that they have no competing interests.

## Authors’ contributions

MS, KG and EMS developed the concept, ran the calculations and drafted the manuscript. AS implemented the spinal cord segmentation. SN gave support for the calculations. PH, JD and RB discussed the outcome at various stages of the project. All authors read and approved the final manuscript.
